# Identification and antibiotic susceptibility pattern of coagulase-negative *staphylococci *in various clinical specimens

**DOI:** 10.12669/pjms.296.4064

**Published:** 2013

**Affiliations:** 1 Muhammad Murad Ehsan, MBBS, Department of Pharmacology, Ziauddin University, Karachi-75600, Pakistan.; 2 Zahida Memon, MBBS, M.Phil, PhD, Department of Pharmacology, Ziauddin University, Karachi-75600, Pakistan.; 3 M.Owais Ismail, MBBS, M.Phil, Department of Pharmacology, Ziauddin University, Karachi-75600, Pakistan.; 4 Ghulam Fatima, MBBS, M.Phil, MCPS, Department of Microbilogy, Central Lab, Civil Hospital, Karachi, Pakistan.

**Keywords:** Antibiotic resistance, Coagulase negative staphylococci, Oxacillin

## Abstract

***Objective: ***Antibiotic resistance is a global problem and is more prevalent in developing countries. Coagulase-negative staphylococci (CoNS) are recognized as important pathogen for nosocomial infections. This study was carried out to identify CoNS in various clinical specimens and to determine its antimicrobial susceptibility pattern.

***Methods: ***A total of 2989 specimens of blood, pus and wound swab were collected from wards, casualty, ICU and OPD, out of these, staphylococci were isolated in 1000 specimens, of which 381 were identified as CoNS. Culture, gram stain, catalase, coagulase test and antimicrobial susceptibility pattern were done according to clinical manual of microbiology. A total of fourteen antibiotics were used in this study. Susceptibility testing was done by Kirby Bauer disc diffusion technique.

***Results: ***Antimicrobial resistance of CoNS were Oxacillin (70.3%), Amoxicillin (74.8%), Amoxicillin+clavulanate (32.8%), Ciprofloxacin (35.2%), Ofloxacin (33.6%), Ceftriaxone (30.4%), Erythromycin (58.3%), Clindamycin (16.3%), Daptomycin (42.5%), Kanamycin (52.2%), Fusidic acid (41.7%), Doxycycline (24.7%), Vancomycin (2.6%) and Linezolid (0.8%). Maximum Oxacillin resistance was between 80 to 90 percent in a group of patients having age of 45 to 65 years and those suffering from cancer or admitted in ICU.

***Conclusion: ***The study concluded that CoNS showed significant level of resistance against most of the widely used therapeutic agents.

## INTRODUCTION

In the past 60 years, antibiotics have been fighting against infectious diseases caused by various bacteria and microbes and have a very important role. However, due to inappropriate and irrational use of antimicrobial drugs, conditions are favorable for resistant microorganisms to emerge, spread and persist.

Wound, pus, and septicemia are just a few of the sources of infections that have become hard to treat with antibiotics. The global problem of antimicrobial resistance is a major concern in developing countries where the infectious disease burden is generally high due to economic factors as infectious diseases are not treated optimally due to limitations in application of expensive drugs.

Multidrug resistant organisms including staphylococci are mainly acquired in hospital settings, where these organisms are treated by a broad spectrum and prolong use of antimicrobials.^,^ Staphylococcal infections may spread through contact with an infected person or with pus from an infected wound, and also spread through contact with personal belongings of patients such as towels, sheets, clothings and athletic equipments. Healthcare workers are also the source of transportation of bacteria by picking them from one patient and pass it on to other patient.

Coagulase negative staphylococci (CoNS) were considered as contaminant organisms in the past but now it is realized in various parts of the world and has become an established fact that it causes infections. Nosocomial bacteraemia is most commonly caused by coagulase-negative staphylococci (CoNS), so it is important to explore the sources of CoNS for prevention and management of infections. Coagulase-negative staphylococci are common colonizers of the human skin and are responsible for most of the infections at this site including abscess and wound infections. These micro organisms are also present in patients with indwelling medical devices such as central and peripheral venous catheters, valvular prostheses, artificial heart valves, pace-makers and orthopedic prostheses where they produce biofilm, which is the source of infection. A number of different CoNS species have been defined, but their identification is not usually necessary as there are no such clear association between the species and types of infection.

Resistance to methicillin and semi synthetic penicillins has been observed in more than 80 percent of coagulase-negative staphylococcal isolates. Such isolates are often resistant to multiple classes of antibiotics in addition to beta-lactams. The genes responsible for resistance are often found on plasmids, facilitating the horizontal exchange of resistance genes among strains. The mecA gene encoding a low-affinity penicillin-binding protein (PBP 2a) is responsible for mediating methicillin or oxacillin resistance in CoNS, as in Staphylococcus aureus.

It has become an established fact that infections caused by coagulase negative staphylococci are on a rise and afflicts most of the population worldwide. Extensive literature survey revealed that CoNS showed resistance against most of the common and frequently used therapeutic antibacterial agents. Therefore, this study was conducted to determine the frequency of CoNS in various infected specimens and antibiotic susceptibility pattern against these microbes.

## METHODS

***Study population and Collection of samples: ***The study was conducted at Civil Hospital Karachi from October 2012 to April 2013. Age range of patients was 18 to 80 years, and was divided into four groups. Young (< 25 years), young adults (25-45 years), middle age (45-65 years) and old age (>65 years). Patients were also divided into five groups on the basis of their clinical diagnosis that are as follows; a) suffering from cutaneous abscess b) post operative complications c) respiratory tract infections d) infections secondary to cancer lesions and e) meningitis. A total of 2989 specimens of blood, pus and wound swabs were collected from wards, casualty, ICU (medical/surgical) and OPD. Staphylococci were isolated in these specimens and CoNS were identified as per protocol. 

The specimens of pus or wound swabs were collected by disposable sterile swab stick. In suspected septicemic patients, 5ml of blood was collected by venepuncture under sterile conditions and was collected in BacTec blood culture bottle. All these samples were transferred to microbiology lab for culture and sensitivity.

***Blood*** ***Culture:***

On day 1, inoculation of 5ml of blood in a liquid media like BD BacTec blood culture bottle, which was placed in BacTec9240 blood culture system after scanning the bar code, and than incubated for five days. 

From day 2 onwards, when the machine beeps for positive growth, bottle was taken out after scanning as positive. It was followed by subculture on blood agar, chocolate agar and MacConkey agar. Plates were incubated at 37^0 ^C aerobically and examined for bacterial growth. Finally, gram stain, catalase and coagulase test were done for identification and Kirby Bauer disc diffusion technique for antibiotic sensitivity.

***Pus and Wound swab Culture:***

On day 1, inoculation of pus and wound swab on blood agar, MacConkey agar and Robertson’s cooked meat medium, followed by incubation at 37^0^ C aerobically overnight. Gram Stain was done to see pus cells and bacteria.

From Day-2 onwards, subculture from cooked meat medium on blood agar, MacConkey agar and incubation of plates at 37^0^ C were done. These plates were examined for bacterial growth. Finally, gram stain, catalase and coagulase test were done for identification and Kirby Bauer disc diffusion technique for antibiotic sensitivity. 

***Species identification: ***Characteristic Staphylococcal colonies were identified. Gram stain, catalase and coagulase testing were done according to clinical manual of microbiology.

***Antibiotic Susceptibility Testing: ***A total of fourteen (14) antibiotics which represent the most commonly used antibiotics in the study area are used in this study. 

Susceptibility testing was done by Kirby Bauer disc diffusion technique. Zones of inhibition were measured around disk and were interpreted according to National Committee for Clinical Laboratory Standards Guidelines (NCCLS).^12^ Susceptibility pattern was noted as sensitive, resistant and intermediate.

All confirmed CoNS isolates were screened for Oxacillin (5μg) Amoxicillin (25μg), Amoxicillin+clavulanate(30μg), Doxycycline(30μg), Ceftrioxone(30μg), Ciprofloxacin(5µg), Ofloxacin(5μg), Clindamycin(2μg), Vancomycin(30μg), Linezolid(30μg), Kanamycin(30μg), Erythromycin (15μg), Fusidic acid(10μg), Daptomycin (30 μg).

***Statistical analysis: ***Data were entered on statistical package of social science (SPSS) version 20. Mean and standard deviation was calculated for numerical variable like age. Frequencies and percentages were calculated for categorical variables like gender, source of specimen, department and type of antibiotics administered. Chi square test was applied to find association between oxacillin resistance with age groups, gender, diagnosis, source of specimen and type of specialty. 

P value was calculated at 95% confidence interval.

## RESULTS

**Fig.1:**
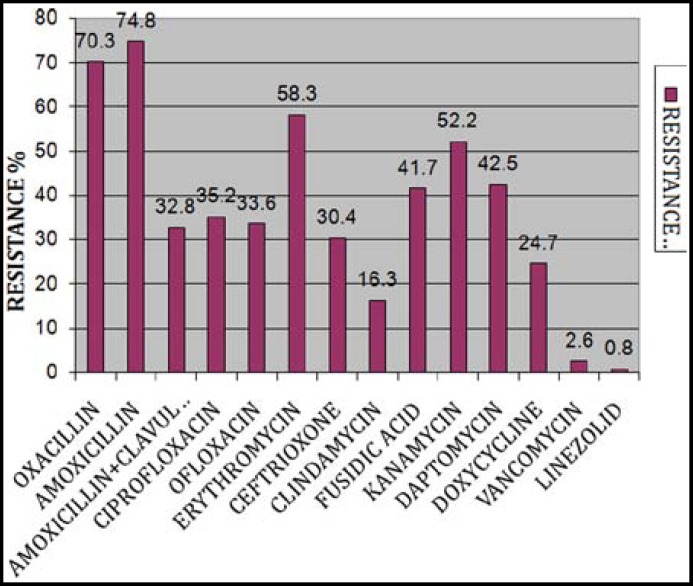
Antibiotic resistance pattern of various CoNS.

**Fig.2:**
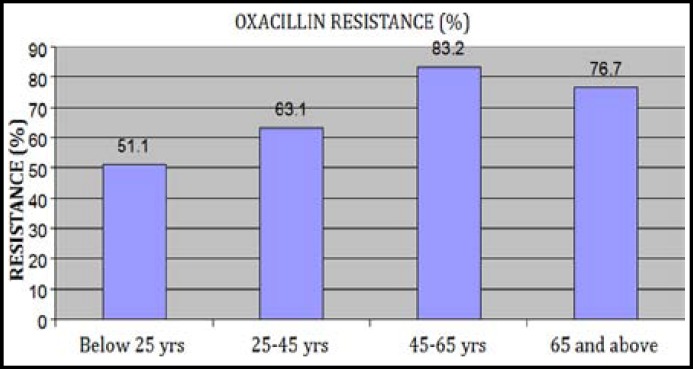
Oxacillin resistance of CoNS in different age groups. P value < 0.05

A total of 2989 specimens were processed and on the basis of our identification methods, 1000 were isolated as staphylococci in these specimens, of which 381 were identified as coagulase negative staphylococci. Most of the isolates were obtained from blood 175 (45.9%), followed by pus 140 (36.7%) and wound swab 66 (17.3%) as shown in Table-I. Antibiotic resistance pattern of CoNS showed that most of the isolates were resistant against Amoxicillin (74.8%) and Oxacillin (70.3%), while least resistance was observed against Vancomycin (2.6%) and Linezolid (0.8%) respectively. Resistance pattern for remaining therapeutic agents were in the range of 16.3% to 58.3% as shown in Fig.1. Oxacillin resistance for patients who were suffering from infections secondary to cancer lesions, meningitis, abscess, post operative complications and respiratory tract infections was found as 88.6%, 85.7%, 71.4%, 65.2% and 62.9% respectively (Table-II) (P-Value< 0.05). The pattern of oxacillin resistance was found to be 83.2%,76.7%, 63.1% and 51.1% in middle age, old age, young adult and young respectively as shown in Fig.2 (P-Value <0.05). Data was also calculated for various groups who have been admitted in different wards. Pattern of oxacillin resistance was 84.4%, 75.9%, 72.0%, 70.0%, 68.8%, 68% and 62.9% in patients who were admitted in ICU, surgical ICU (SICU), medicine, orthopedics, surgery, OPD and casualty respectively (Table-III), which showed non-significant association of oxacillin resistance with various aforementioned groups.

**Table-I:** Frequencies of CoNS in different specimens.

*Specimen*	*Frequency N (%)*
Blood	175(45.9)
Pus	140(36.7)
Wound Swab	66(17.3)
Total	381(100.0)

**Table-II:** Frequencies of CoNS in patients suffering from different infections.

*Diagnosis*		*Oxacillin*		*Total N (%)*
	*Intermediate* *N (%)*	*Resistant* *N (%)*	*Sensitive* *N (%)*	
Abscess	1(0.8)	*90(71.4)	35(27.8)	126((33.1)
Post operative complications	1(1.5)	*43(65.2)	22(33.3)	66(17.3)
Respiratory tract infection	0(0.0)	*78(62.9)	46(37.1)	124(32.5)
Infections sec. to Cancer lesions	0(0.0)	*39(88.6)	5(11.4)	44(11.5)
Meningitis	0(0.0)	*18(85.7)	3(14.3)	21(5.5)
Total	2(0.5)	268(70.3)	111(29.1)	381(100.0)

**Table-III:** Frequencies of various CoNS, obtained from different departments.

*Departments*		*Oxacillin*		*Total*
	*Intermediate* *N (%)*	*Resistant* *N (%)*	*Sensitive* *N (%)*	*N (%)*
Casualty	0(0.0)	*44(62.9)	26(37.1)	70(18.4)
O.P.D	0(0.0)	*34(68.0)	16(32.0)	50(13.1)
Medicine	0(0.0)	*72(72.0)	28(28.0)	100(26.2)
Surgery	2(2.5)	*55(68.8)	23(28.8)	80(21)
Orthopedics	0(0.0)	*14(70.0)	6(30.0)	20(5.2)
I.C.U	0(0.0)	*27(84.4)	5(15.6)	32(8.4)
S.I.C.U	0(0.0)	*22(75.9)	7(24.1)	29(7.6)
Total	2(0.5)	268(70.3)	111(29.1)	381(100.0)

* = P value >0.05

## DISCUSSION

Despite the introduction of various antimicrobial agents, antibiotic resistance is increasing day by day. It is more prevalent in developing countries due to their misuse. It has been realized worldwide that most of the nosocomial infections are caused by CoNS. In our study 381 CoNS are isolated from various clinical specimens including blood (45.9%), pus (36.7%) and wound swab (17.3%), which is nearly similar to the study done in Iran and Tunisia in which number of CoNS isolates in blood were 25.4% and 29% respectively.

However results were conflicting with the Indian studies done by Habeeb and Mohan^,^ respectively, which showed higher number of CoNS isolates in pus. Although this high incidence reported in pus cannot be explained scientifically and also not supported by international literature but it might be due to higher number of wound infections in postoperative patients possibly secondary to either improper sterilization of instruments during operation or inadequate hygienicity. Susceptibility pattern of CoNS in our study showed that the resistance of CoNS against oxacillin is (70.3%) that is nearly similar to the study done at China, U.S.A, Turkey and India. Also oxacillin resistance in various parts of the Europe is in the range of 70% to 80%.^,^ Our data also showed that patients who were admitted in I.C.U, showed high oxacillin resistance, which is 84.4% as compare to the patients admitted in other departments. This is nearly similar to the study done at Brazil (81.6%) and Karolinska (92%) in which the resistance against oxacillin is also high in patients who were admitted in I.C.U. The higher prevalence of oxacillin resistance in the I.C.U is probably due to invasive procedures, critical conditions and prolong stay of the patients.

In the present study, oxacillin resistance of 83.2% was observed in a group of patients having age of 45 to 65 years, which is higher than the resistance in other age groups. Also patients who were suffering from infections secondary to cancer lesions showed highest oxacillin resistance of 88.6%. It is therefore concluded that the chances of resistance against oxacillin and to other conventional antibiotics is higher in a patients either suffering from cancer or admitted in I.C.U and those having age of 45 to 65 years, which is a real threat for these patients in terms of treating their infections.

## CONCLUSION

The emergence of antibiotic resistance against coagulase negative staphylococci is a matter of serious concern in our set of population that is strongly supported by many international latest research findings. Present study showed high resistance of coagulase negative staphylococci against oxacillin and other commonly used therapeutic agents. This increasing oxacillin resistance is gradually becoming a threat for various multi drug resistant bacteria in clinical settings. Therefore regular surveillance of antibiotic susceptibility against CoNS in a hospital should be done and its irrational use should be avoided in order to control the spread of infections and for better management of infectious diseases.

## Notes

***Source of funding:*** Ziauddin University.
